# Differential insular cortex subregional vulnerability to α‐synuclein pathology in Parkinson's disease and dementia with Lewy bodies

**DOI:** 10.1111/nan.12501

**Published:** 2018-06-26

**Authors:** Y. Y. Fathy, A. J. Jonker, E. Oudejans, F. J. J. de Jong, A.‐M. W. van Dam, A. J. M. Rozemuller, W. D. J. van de Berg

**Affiliations:** ^1^ Section Clinical Neuroanatomy Department of Anatomy and Neurosciences Amsterdam Neuroscience VU University Medical Center Amsterdam The Netherlands; ^2^ Department of Neurology Erasmus Medical Center Rotterdam The Netherlands; ^3^ Department of Pathology Amsterdam Neuroscience VU University Medical Center Amsterdam The Netherlands

**Keywords:** alpha synuclein, astrocytes, insular cortex, Parkinson's disease, von Economo neurons, vulnerability

## Abstract

**Aim:**

The insular cortex consists of a heterogenous cytoarchitecture and diverse connections and is thought to integrate autonomic, cognitive, emotional and interoceptive functions to guide behaviour. In Parkinson's disease (PD) and dementia with Lewy bodies (DLB), it reveals α‐synuclein pathology in advanced stages. The aim of this study is to assess the insular cortex cellular and subregional vulnerability to α‐synuclein pathology in well‐characterized PD and DLB subjects.

**Methods:**

We analysed postmortem insular tissue from 24 donors with incidental Lewy body disease, PD, PD with dementia (PDD), DLB and age‐matched controls. The load and distribution of α‐synuclein pathology and tyrosine hydroxylase (TH) cells were studied throughout the insular subregions. The selective involvement of von Economo neurons (VENs) in the anterior insula and astroglia was assessed in all groups.

**Results:**

A decreasing gradient of α‐synuclein pathology load from the anterior periallocortical agranular towards the intermediate dysgranular and posterior isocortical granular insular subregions was found. Few VENs revealed α‐synuclein inclusions while astroglial synucleinopathy was a predominant feature in PDD and DLB. TH neurons were predominant in the agranular and dysgranular subregions but did not reveal α‐synuclein inclusions or significant reduction in density in patient groups.

**Conclusions:**

Our study highlights the vulnerability of the anterior agranular insula to α‐synuclein pathology in PD, PDD and DLB. Whereas VENs and astrocytes were affected in advanced disease stages, insular TH neurons were spared. Owing to the anterior insula's affective, cognitive and autonomic functions, its greater vulnerability to pathology indicates a potential contribution to nonmotor deficits in PD and DLB.

## Introduction

Parkinson's disease (PD) is mainly characterized by motor symptoms which result from the death of dopaminergic neurons in the substantia nigra pars compacta 1. Yet, nonmotor deficits, including cognitive impairment, autonomic dysfunction and neuropsychiatric symptoms are highly prevalent in PD 2, 3, 4. In addition, dementia with Lewy bodies (DLB), one of the most common causes of dementia, is defined by an early onset of fluctuating cognition, visual hallucinations and dementia preceding or occurring concomitantly within 1 year from the onset of parkinsonism 5, 6. PD with dementia (PDD) and DLB show considerable clinical overlap and may be considered as two ends of a disease spectrum with different timing of parkinsonism and dementia 7, 8, 9. In PD and DLB, the catecholaminergic, dopaminergic and nor‐adrenergic nuclei in the brainstem and cortex are particularly vulnerable to α‐synuclein pathology and degeneration 10. Loss of these monoaminergic neurons most likely contribute to the cognitive and neuropsychiatric deficits in these disorders 10, 11. It is therefore imperative to study the regional and cellular correlates of clinical deficits in PD and DLB. Of interest in this respect, the insular cortex is involved in the integration of somatosensory and autonomic information with higher cognitive functions 12, 13, 14. It also plays a role in emotion recognition, cognition and awareness of interoceptive information and thus acts as the basis of self‐awareness 15. It has been associated with multiple neuropsychiatric disorders, such as anxiety, depression and bipolar disorder 16. However, little is known regarding the selective vulnerability of catecholaminergic and other cells in this region in PD and DLB.

According to Braak staging for PD, α‐synuclein pathological aggregates progress from brainstem to limbic brain regions in the prodromal and early stages of the disease followed by the neocortex in more advanced stages. Meanwhile, the insular cortex is affected in advanced stages of the disease (5 and 6) 17, 18, 19. Atrophy of the insula in PD, assessed by neuroimaging, has also been associated with executive dysfunction, one of the most common and early cognitive dysfunctions in PD 20. Moreover, a reduction in dopaminergic receptor binding and grey matter density have been associated with mild cognitive impairment in PD 21, 22. Insular atrophy was also found in patients with prodromal DLB and correlates with impairment in attributing mental states to others in patients with probable DLB 23, 24.

**Figure 1:**
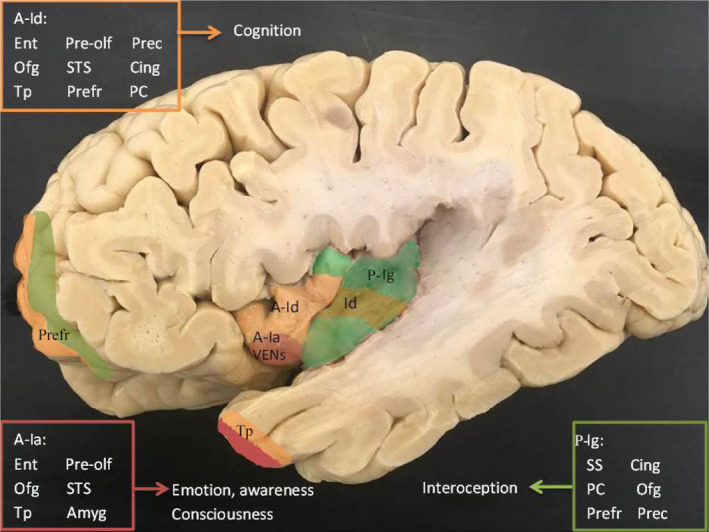
Macroscopy of the insular cortex subregions and corresponding connections. The insular cortex is seen within the sylvian fissure. The agranular insula (A‐Ia) is seen ventro‐anteriorly (red), is connected to the olfacotory cortex, orbitofrontal, amygala and temporopolar region. While the dysgranular insula (A‐Id) is seen dorsally (orange) and is connected to various limbic and neocortical regions. Although the granular insula (P‐Ig) (green) is mostly present within the posterior insula. It is preferentially connected to the somatosensory cortex, parietal cortex and cingulate. Some regions are coloured to outline connections to the insular subregions including the prefrontal cortex and temporopolar cortex. Amyg, amygdala; Cing, cingulate gyrus; Ent, entorhinal cortex; Ofg, orbitofrontal gyrus; PC, parietal cortex; Prec, precentral sulcus; Prefr, prefrontal cortex; Pre‐olf, prepiriform part of olfactory cortex; SS, somatosensory cortex; STS, superior temporal sulcus; Tp, temporopolar cortex.

Anatomically, the insula is a heterogeneous region hidden deep within the Sylvian fissure and is widely connected to the brain. Macroscopically, the insula is divided into anterior and posterior gyri both constituting different cytoarchitectures. Microscopically and in order from ventro‐rostral to dorso‐caudal, the main subregions are defined as anterior periallocortical agranular (Ia), anterior‐ middle pro‐isocortical dysgranular (Id), and posterior isocortical granular (Ig) and hypergranular (G) subregions, based on the cytoarchitecture and number of layers 25, 26, 27, 28. According to the location and connectivity, the agranular insula mostly relays projections to limbic regions and the granular insula mostly projects to cortical areas 12, 29. Preferential projections to the anterior insula arise from the prepiriform olfactory, orbitofrontal and rhinal cortices. On the other hand, only the posterior insula receives projections from the secondary somatosensory areas. The dysgranular insula represents a transitional zone with a variety of limbic and cortical connections 29, 30 (Figure 1). On the basis of the current staging criteria and earlier involvement of the limbic cortex compared to the neocortex in PD, the diversity in cytoarchitecture of the insular subregions could provide insight into the underlying characteristics predisposing to degeneration. Moreover, the presence of von Economo neurons (VENs), spindle shaped neurons in layer Vb, mostly in the agranular insula, adds to the uniqueness of the region. VENs are implied to play a role in social awareness, emotional processing and autonomic control 31, 32. Despite speculations on their role in cognitive decline in disease 23, it remains unknown if VENs are vulnerable to α‐synuclein pathology in PD and DLB. Moreover, the selective vulnerability of catecholaminergic neurons in the insular cortex subregions remain unknown.

Considering the wide‐spread connectivity of the insula, the cellular heterogeneity and differential functional properties of the insular subregions, we hypothesize that the periallocortical agranular subregion of the insula displays greater vulnerability to α‐synuclein pathology in PD and DLB compared to the isocortical subregions. To gain insight into the selective vulnerability of the insular subregions and their cell types, we performed a detailed analysis of the α‐synuclein distribution pattern throughout the insular cortex of subjects with incidental Lewy body disease (iLBD), PD and DLB. Our study provides data on the selective vulnerability of VENs, catecholaminergic neurons and astrocytes within the insular subregions.

## Materials and methods

### Post mortem human brain tissue

Insular post mortem tissues from 21 donors with iLBD, PD(D) and DLB (range = 60–93 years) and 3 age‐matched controls (range = 68–79 years) were collected by the Netherlands Brain Bank (www.nbb.nl; Netherlands) and the Normal Aging Brain Collection Amsterdam (www.nabca.eu; Netherlands). All donors had provided written informed consents for donation of brain tissue and access to clinical and neuropathological reports in compliance with ethical and legal guidelines. The main inclusion criteria were: (i) clinical diagnosis of PD(D) or DLB according to revised MDS diagnostic criteria 6, 33 and (ii) pathological confirmation of diagnosis 34. Subjects were excluded if they had a long history of neuropsychiatric disorders or suffered from insular infarcts.

The entire insula was dissected into 0.5–1 cm thick blocks and defined according to its borders with orbitofrontal and temporal cortices inferiorly and inferior frontal gyrus operculum superiorly 35. Tissue blocks were cryo‐protected with 30% sucrose, frozen and stored at −30°C until further processing. The tissue slices were then sectioned, using a sliding microtome, into 60 μm thick sections.

### Neuropathological assessment

For neuropathological diagnosis and staging, 6 μm paraffin sections from several brain regions of all donors were stained for α‐synuclein, β‐amyloid, hyperphosphorylated tau, haematoxylin and eosin (H&E), α‐synuclein, TDP‐43 and congo‐red according to current diagnostic guidelines of BrainNet Europe 34. Confirmation of either iLBD, PD or DLB and concommitant Alzheimer's disease (AD) pathology was based on guidelines using Braak staging for neurofibrillary tangles (Braak NFT 0–6), Braak α‐synuclein (Braak α‐syn 0–6), Thal phase for β‐amyloid (0–5), and ABC scoring system 17, 36, 37, 38, 39. Glial tauopathy such as age related tauopathy of the astroglia (ARTAG) and primary age related tauopathy were assessed primarily in the temporal cortex, olfactory cortex and amygdala 40, 41.

### Immunohistochemistry

For α‐synuclein immunostaining, free‐floating 60 μm sections were pretreated with 98% formic acid (Sigma‐Aldrich, Darmstadt, Germany) and incubated with primary antibody mouse anti‐α‐synuclein (1:2000; 610786; BD Biosciences, Berkshire, UK), as previously described 42. Adjacent sections were pretreated with citrate buffer pH 6.0 in a steamer (95°C) and stained using antibodies against tyrosine hydroxylase (TH) antibody (rabbit anti‐TH, 1:1000, incubation for 24 h; AB152, Merck Millipore, Darmstadt, Germany) or astrocytic marker glial fibrillary acidic protein (GFAP) (rabbit anti‐GFAP 1:4000, incubation for 72 h; Z0334; DAKO, Glostrup, Denmark). The sections were incubated in the secondary antibody biotinylated IgG (1:200, Vector Laboratories, Burlingame, CA, USA) followed by standard avidin‐biotin complex (1:200, Vectastatin ABC kit, Standard; Vector Laboratories) in TBS or rabbit Envision (DAKO) for 2 h. Then 3,3′‐diaminobenzidine (DAB) was used to visualize staining and sections were mounted and counter‐stained with thionin (0.13%, Sigma‐Aldrich, Darmstadt, Germany). For double staining of TH and α‐synuclein, liquid permanent red (DAKO) and DAB were used.

For immunofluorescent double staining of α‐synuclein and GFAP, immunostaining was performed as above for 72 h at 4°C followed by incubation with donkey anti‐mouse coupled with Alexa Fluor 488 (1:400; Molecular Probes, Waltham, MA, USA), donkey anti‐rabbit coupled with Alexa Fluor 594, and diamidino‐2‐phenylindole(4,6)dihydrochloride (DAPI; Sigma) for 2 h. The tissue sections were then mounted on glass slides and cover‐slipped with mowiol as mounting medium (4‐88 Calbiochem).

### Bright‐field and confocal laser scanning microscopy

Digital images of the immuno‐stained slides were made with a photomicroscope (Leica DM5000) equipped with colour camera DFC450, Leica LASV4.4 software and 63× oil objective lens. Immunofluorescent labelling was visualized using confocal laser scanning microscopy (CLSM) LEICA TCS SP8 (Leica Microsystems, Jena, Germany). Image acquisition was done using 100×/1.4 NA objective lens, 405 nm diode, and pulsed white light laser (80 Hz) with excitation wavelengths 405, 499 and 598 nm. Afterwards, deconvolution of image stacks was performed using Huygens Professional software (Scientific Volume Imaging, Hilversum, the Netherlands). Colocalization analyses to assess the co‐occurrence and correlation of GFAP and α‐synuclein were performed using Imaris software 8.3 (Bitplane, South Windsor, CT, USA). Deconvolved fluorescent images acquired using CLSM were used and a region of interest (ROI) was outlined for colocalization. The correlation between both channels was determined using Pearson's correlation coefficient and Mander's overlap coefficient (MOC) as well as the percentage of ROI colocalized (http://www.bitplane.com/imaris/imariscoloc).

### Definition of insular subregions

**Figure 2:**
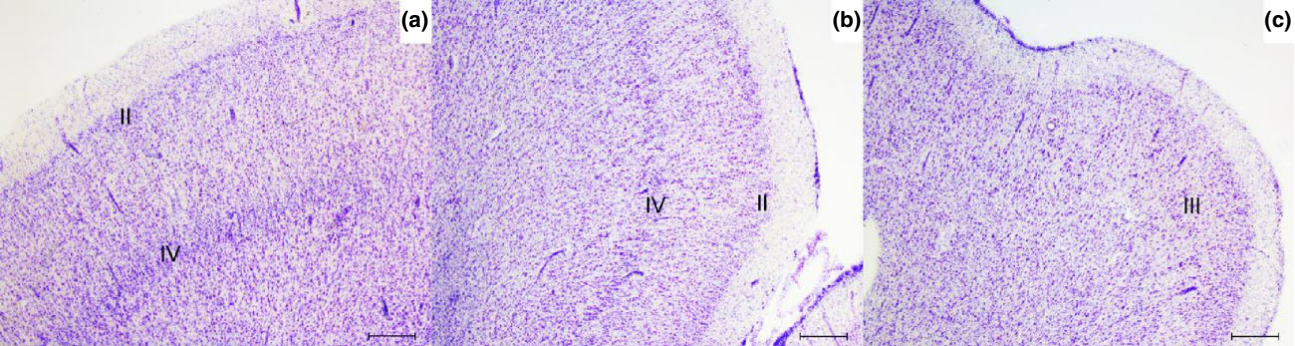
Definition of insular subregions in 60 μm thick sections. (a) Granular insular grey matter shows uniform and well defined granular layers II and IV in an iLBD case. (b) Dysgranular insula shows less dense and granular layers II and IV. (c) Agranular insula grey matter is shown lacking layers II and IV. iLBD, incidental Lewy body disease; II, layer two; III, layer three; IV, layer four. Magnification: 25 ×, scale bar 500 μm.

The anatomical and cytoarchitectural characteristics of the insular subregions were identified in Nissl stained sections by YF and WvdB based on the granularity and density of layers II and IV. For simplicity, definitions were based on the four known subregions: agranular, dysgranular and granular/hypergranular insula 12. The agranular insula was defined based on its ventral anterior location, absence of layers II and IV, and clusters of VENs in layer Vb. The dysgranular region is dorsal to the agranular and has more distinguished layers II and IV. The granular and hypergranular regions were defined based on their dorso‐caudal and mid to posterior location and consisted of increasingly dense and granular layers II and IV 28 (Figure 2). VENs and fork cells were assessed in layer V of the agranular insula and VENs were defined based on their spindle‐shaped morphology and anatomical location 43. α‐Synuclein inclusions in the insular cortex were assessed using an ordinal semiquantitative score, ranging from 0 to 3 in 60 μm sections. The scoring criterium was: 0 = absent, 1 = few dot‐like deposits or sparse LNs present + 1–5 LB, 2 = Moderate LN present in all layers + 5–10 LB, 3 = Severe LNs present in all layers + >10 LB in 20× objective field. α‐synuclein deposits were counted in all layers and ≥3 frames. The subregions were first defined and the scoring was performed by YF and EO as described (Table S1). Kruskal–Wallis test was conducted to evaluate differences in the distribution pattern of α‐synuclein pathology between the three insular subregions (agranular, dysgranular, and granular) and across groups (Figure S1). Statistical significance was set at 0.05.

### Quantification of the density of TH‐immunoreactive neurons in insular subregions

In total, 12 PD(D) and DLB subjects, from which the entire insula was available, were included for quantitative analysis of the TH immunoreactive (TH‐ir) neuronal density (neurons/mm^2^) in the agranular and dysgrnaular subregions. The granular insula did not reveal TH‐ir neurons and was not included in the density assessment. The insular subregions were defined and all layers were evaluated by YF and EO for presence of TH‐ir neurons. A region of interest (ROI) was then defined at 25× magnification from midlayer III to midwhite matter layer where most TH‐ir neurons were located, using the stereoinvestigator software (11.06.200; MBF Bioscience, Delft, The Netherlands). TH‐ir neurons had a diameter range from 7 to 29 μm, consistent with data available in the literature 44. As only few TH‐ir neurons were observed in the insular subregions, we determined the local density in the agranular and dysgranular subregions within the anterior insula in one section per case. The TH‐ir neurons were included in the density assessment when they met the following criteria: (i) soma with diameter >7 μm; (ii) (part of) neurites visible; (iii) soma was located within or intersecting the lines of the ROI. TH‐ir neurons were then counted at 400× using the Meander scan. The local density of TH‐ir neurons (neurons/mm^2^) was calculated per subregion for each case as previously described by others 10.

### Statistical analysis

SPSS version 22 (IBM, Armonk, New York, USA) was used for all statistical analyses. One‐way ANOVA (multiple group comparison) with Bonferroni *post hoc* test was used to examine differences in subject demographics between groups. The Kruskal–Wallis test was used to compare the TH density of the agranular and dysgranular insula. Differences in TH density per subregion between the PD, PDD, DLB patients and controls were analyzed using one‐way ANOVA. Furthermore, the correlation between TH density per subregion and α‐synuclein was examined using Spearman's rho. Statistical significance was set at 0.05.

## Results

### Clinical and neuropathological characteristics of PD and DLB donors

All PD(D) and DLB donors included in this study had Braak α‐synuclein stages ranging from III to VI and disease duration ranged from 2 to 26 years. The PDD and DLB donors had advanced Braak α‐synuclein stages (5–6), moderate to severe cognitive impairments with memory, attention, language problems and REM sleep behavioural disorder. Several neuropsychiatric symptoms were reported including visual hallucinations, delirium, depression, anxiety and panic attacks. Moreover, ARTAG was present in all PDD cases except for PDD_3 who showed severe astroglial degeneration instead. NFT and β‐amyloid plaques were most severe in DLB‐1 and DLB‐2 who had a short disease duration (2–3 years) as well as a family history of AD and early dementia. The demographics and neuropathological staging of all donors included in this study are summarized in Table 1.

**Table 1:** Subject demographics and neuropathological staging

Subject ID	Gender	Age death (year)	Diagnosis	Age at onset (year)	DD	Braak α‐syn	Braak NFT & tauopathy	Thal phase	ABC	Cognitive and psychiatric deficits
HC										
HC_1	M	68	Control	N/A	N/A	0	I, ARTAG	2	A1B1C0	N/A
HC_2	M	74	Control	N/A	N/A	0	II	3	A2B1C1	N/A
HC_3	F	79	Control	N/A	N/A	0	II	3	A2B1C0	N/A
iLBD and PD										
iLBD‐1	F	88	iLBD	N/A	N/A	4	III	2	A2B2C1	N/A
PD‐1	M	78	PD	75	3	3	I	0	A0B1C0	N/A
PD‐2	F	93	PD	91	2	3	II	2	A1B1C0	Depression
PD_3	M	77	PD	66	11	5	II	1	A1B1C0	Word‐finding difficulties, poor attention & concentration
PD_4	F	68	PD	52	16	5	II	1	A1B1C0	MCI
PD_5	F	73	PD	70	3	4	II	2	A1B1C0	Anhedonia and apathy
PDD										
PDD‐1	F	88	PDD	73	15	5	II, ARTAG and PART	0	A0B1C0	Delirium, anxiety, RBD, & hallucinations
PDD‐2	M	74	PDD	67	7	6	II, ARTAG	3	A2B1C0	Depression, panic attacks, & hallucinations
PDD‐3	F	74	PDD	61	13	6	III	3	A2B2C1	Delirium and hallucinations
PDD‐4	F	81	PDD	73	8	6	II, ARTAG	3	A2B1C1	Delirium, hallucinations, & MCI
PDD‐5	M	75	PDD	69	6	6	II, ARTAG	1	A1B1C0	RBD, depression, MCI
PDD‐6	M	70	PDD	51	19	6	III, ARTAG	3	A2B2C0	Cognitive impairment and delirium
PDD‐7	F	88	PDD	82	6	6	II, ARTAG	1	A1B1C0	Hallucinations & memory complaints
PDD_8	M	81	PDD	63	18	6	III, ARTAG, PART	0	A0B2C0	Delirium, hallucinations, anxiety
PDD_9	F	83	PDD	69	14	6	IV, ARTAG, PART	0	A0B2C0	Delirium, hallucinations, dementia
PDD_10	M	71	PDD	45	26	5	II, ARTAG	1	A1B1C0	Hallucinations, delirium, disturbed speech & concentration
DLB										
DLB‐1	M	67	DLB	64	3	6	V, ARTAG	5	A3B3C3	RBD, hallucinations, apraxia, & Capgras syndrome
DLB‐2	M	75	DLB	73	2	6	III, ARTAG	4	A2B2C3	Paranoia, psychosis, hallucinations & Charles Bonnet syndrome
DLB‐3	M	81	DLB	77	4	6	III	3	A2B2C0	RBD, anxiety, depression, disinhibition, & hallucinations
DLB_4	M	78	DLB	72	6	6	I, ARTAG	3	A2B1C0	Memory complaints & Dementia
DLB_5	M	60	DLB	53	7	6	0	0	A0B0C0	Impaired memory, language, concentration, & psychosis

ABC score, A–C (0–3); ARTAG, ageing‐related tau astrogliopathy; Braak NFT, 0–6; Braak α‐syn, 0–6; DLB, dementia with Lewy bodies; DD, disease duration; HC, Healthy control; iLBD, incidental Lewy body disease; MCI, mild cognitive impairment; NFT, neurofibrillary tangles; PART, primary age‐related tauopathy; PD, parkinson's disease; PDD, PD dementia; RBD, REM sleep behavioural disorder; Thal phase, 0–5; α‐syn, α‐synuclein; N/A, not applicable.

### Distribution pattern of α‐synuclein pathology in the insular cortex of iLBD, PD and DLB

We observed a decreasing gradient of α‐synuclein pathology load from the ventral anterior agranular subregion to the dorsal dysgranular and posterior dorso‐caudal granular subregions. In iLBD and PD(D), α‐synuclein deposits were present in all layers of the agranular insula, whereas in the dorsal dysgranular less immunoreactivity was observed. In the granular insula, α‐synuclein immunoreactivity was minimal or absent. LNs were present in all layers while LBs were predominantly found in the deep layers V and VI.

**Figure 3:**
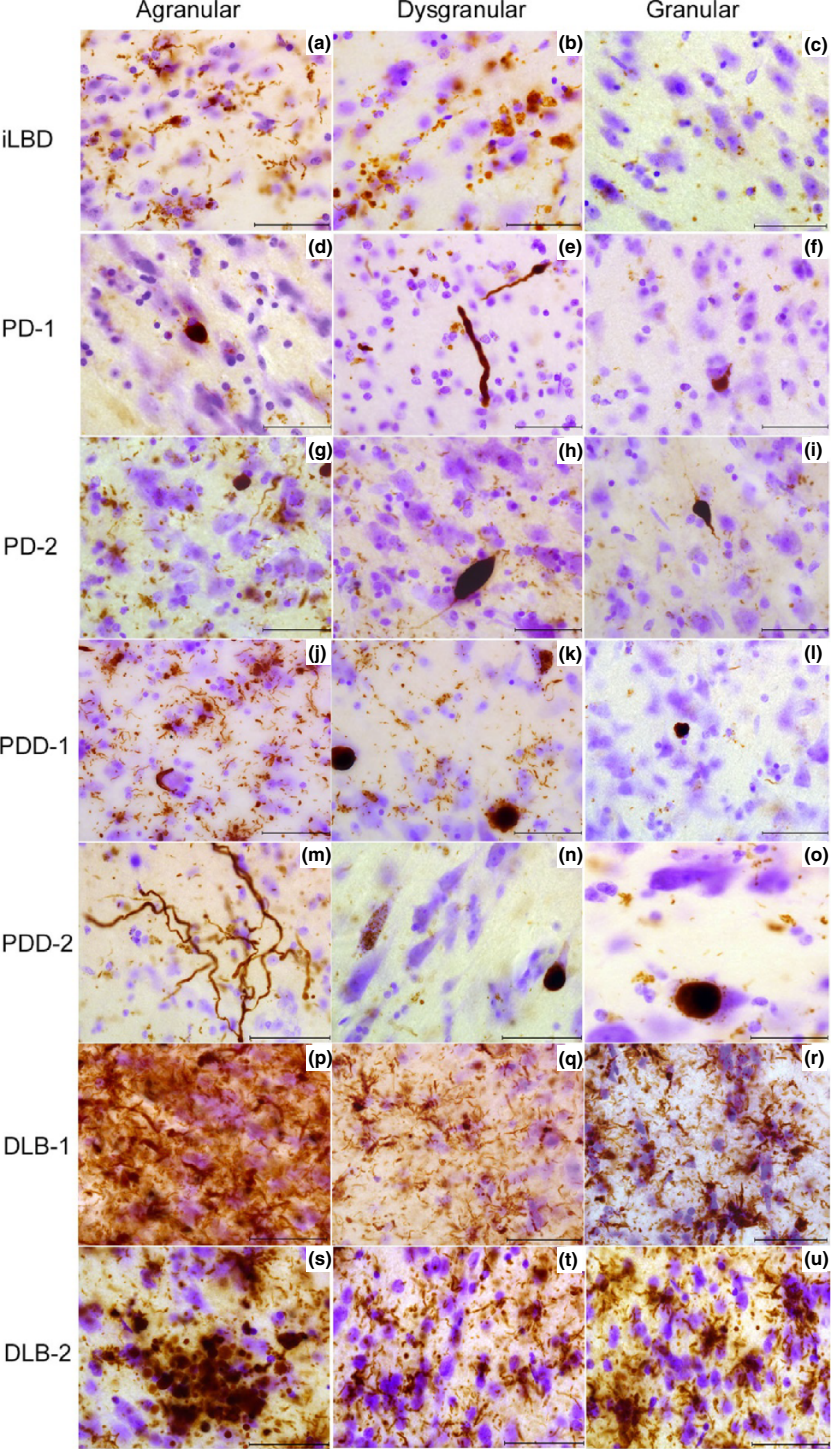
Distribution pattern of α‐synuclein in insular subregions. iLBD shows mild LNs and astroglial α‐synuclein inclusions in layer I of agranular insula (**a**), few glial inclusions in dysgranular insula (**b**), and sparse dot‐like aggregates in granular insula (**c**). PD‐1 agranular insula shows a LB‐like inclusion and dot‐like aggregates (**d**), the dysgranular insula shows bulgy LNs in layer I (**e**), and the granular insula shows an intracellular LB inclusion (**f**). PD‐2 shows many LNs inclusions in agranular insula and glial α‐synuclein (**g**) and less but bulgy LN in dysgranular (**h**) and granular regions (**i**). In PDD‐2 severe astroglial α‐synuclein inclusions are shown in agranular insula (**j**) few LBs and LNs in dysgranular insula (**k**). The granular insula shows dot‐like aggregates and astroglial α‐synuclein **(l)**. In PDD‐1 agranular insula, very long LNs and some dot‐like aggregates are seen in layer I (**m**). Dysgranular insula in PDD‐1 shows granular cytoplasmic inclusions in neurons and a LB (**n**) while the granular insula shows less aggregates and a LB in the infragranular layer (**o**). In DLB‐1, severe α‐synuclein inclusions are seen in agranular insula throughout all layers (**p**). Severe astroglial inclusions are seen in the supragranular layers of dysgranular and granular insula (**q**,**r**). In DLB‐2, a cluster of dystrophic LNs and glial inclusions are shown in layer II of the agranular insula (**s**). The dysgranular insula contains LNs and dot‐like structures (**t**) also abundant in the granular insula superficial layers (**u**). DLB, dementia with Lewy bodie; iLBD, incidental Lewy body disease; LB, Lewy bodies; LN, Lewy neurites; PD, Parkinson's disease; PDD, Parkinson's disease dementia. Magnification: 630 × , scale bar 50 μm.

The iLBD insular cortex (iLBD‐1), Braak α‐synuclein stage 4/6, showed few LN in both agranular and dysgranular regions and very mild immunoreactivity in the granular insula. α‐Synuclein immunoreactive features consisted of dot‐like deposits, few LNs, sparse LBs and astroglial deposits (Figure 3 **a**–**c**). PD‐1, Braak α‐synuclein stage 3/6, revealed very sparse α‐synuclein inclusions in all subregions (Figure 3 **d**–**f**). PD‐2, Braak α‐synuclein stage 3/6, showed moderate to severe LN and few LBs in the deep layers of the agranular insula. The granular and dysgranular subregions contained bulgy LNs as well as a mild to moderate number of LNs and LBs, respectively (Figure 3 **g**–**i**).

In PDD 1‐2, Braak α‐synuclein stage 6/6, the same gradient of α‐synuclein immunoreactivity was present with highest load of pathology in agranular insula. PDD‐1 showed very long LNs in the superficial layers of the agranular insula and a mild to moderate α‐synuclein load in the granular and dysgranular insula, respectively. Astroglial α‐synuclein inclusions were also found (Figure 3 **j–o**). Other PDD cases showed similar distribution of pathology throughout the subregions as well as astroglial synucleinopathy and degeneration.

In contrast, DLB‐1 and 2, Braak α‐synuclein stage 6/6, showed severe α‐synuclein inclusions in all layers of the agranular/dysgranular insula and severe protoplasmic astroglial α‐synuclein inclusions. In the granular insula, a high load of α‐synuclein inclusions with relative sparing of layers III/IV was observed (Figure 3 **p**–**u**). Other DLB cases showed α‐synuclein distribution similar to PD(D).

**Figure 4:**
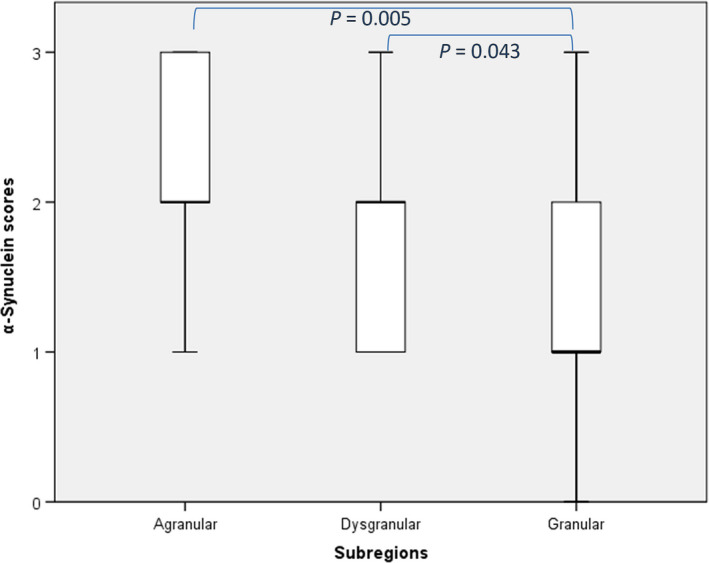
Semiquantitative analysis of α‐synuclein pathology across the insular subregions. The local density of α‐synuclein wass assessed at 200 ×  magnification. A significant difference in subregional distribution of α‐synuclein pathology was observed [χ^2^ (2, *N* = 63) = 9099, *P* = 0.011]. Pairwise comparison between different subregions showed a significant difference between the agranular and granular subregions as well as dysgranular and granular subregions (*P *= 0.005 and 0.043, respectively).

Assessment of the semiquantitative scores of α‐synuclein between the three insular subregions showed a significant difference [χ^2^ (2, *N* = 63) = 9,099, *p *=* *0.011]. To assess pairwise differences across the three subregions, follow‐up tests were performed. Pairwise comparisons between the subregions showed further significant differences between the agranular and granular subregions as well as dysgranular and granular subregions (*P *=* *0.005 and 0.043, respectively) (Figure 4).

### Morphology of α‐synuclein immunoreactive structures in the insular subregions

**Figure 5:**
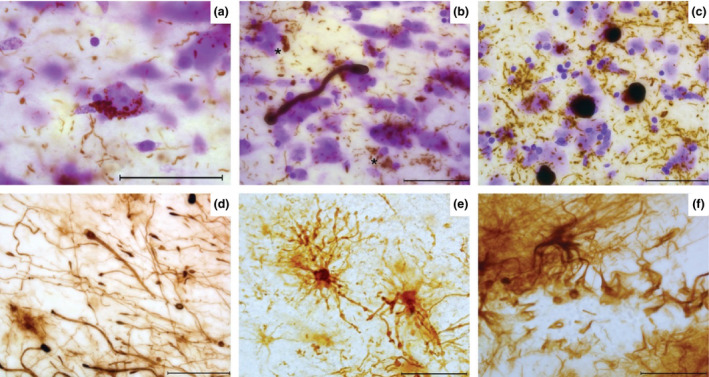
Cell specific morphology and inclusions in Lewy body diseases. In PDD‐2 with astroglial tauopathy, infragranular layer of agranular insula show astroglial‐to‐neuronal α‐synuclein inclusions (**a**), an elongated α‐synuclein positive process with bulbous endings (possibly glial) surrounded by astroglial α‐synuclein inclusions (*) and LNs (**b**). DLB‐2 shows LB inclusions, LNs and astroglial α‐synuclein (*) within the deep infragranular layers (**c**). Loose GFAP + astrocytic processes are shown containing bulbous end feet and donut‐shaped structures in the supragranular layers of the agranular insula in DLB‐2 (**d**). PDD‐1 with astroglial tauopathy shows small and dysmorphic astrocytes containing multiple varicosities within their processes, possibly representing fuzzy astrocytes (**e**). A GFAP + astrocyte is shown surrounded by disorganized processes in DLB‐2 (**f**). Magnification: 630 × , scale bar 50 μm. DLB, dementia with Lewy bodie; LB, Lewy bodies; LN, Lewy neurites; PDD, Parkinson's disease dementia; GFAP, glial fibrillary acidic protein.

In PDD and DLB, severe synucleinopathy was observed in astrocytes (Figure 5 **a**–**c**). The supragranular layers I–III, showed moderate to severe LNs variable in shape and size, thread‐like, bulgy and long. Layers V and VI contained a predominance of cortical LBs, which increased in gradient from agranular to granular subregions. Astroglial degenerative changes in the form of detached astroglial processes with bulbous and doughnut‐shaped end‐feet were present in superficial layers in P DD and DLB. Fuzzy astrocytes, with granular accumulations along their processes were also seen in PDD with ARTAG (Figure 5 **d**–**f**).

### Neuronal vulnerability to α‐synuclein pathology in insular subregions

**Figure 6:**
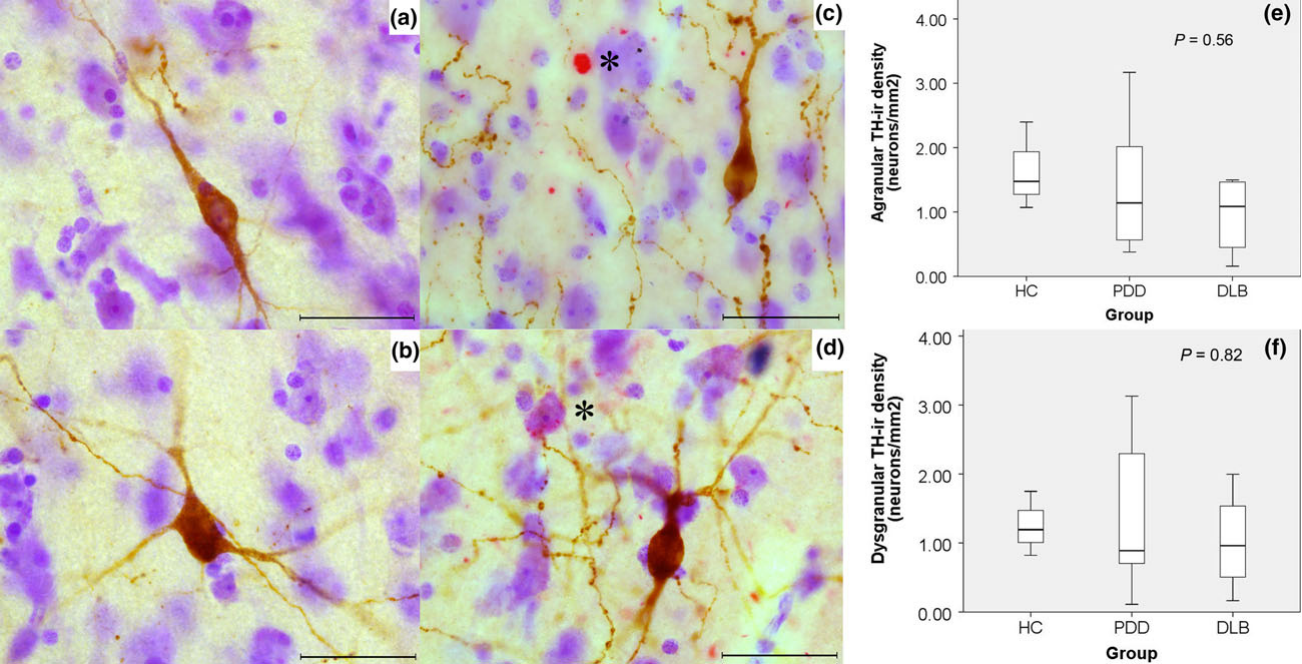
Morphological characteristics of tyrosine hydroxylase immunoreactive (TH‐ir) neurons, distribution pattern, and relationship with α‐synuclein deposits in the Insular cortex subregions. TH‐ir neurons were predominant in layers V and VI, and were mostly bipolar in morphology and few multipolar (**a**,**b**). No α‐synuclein deposits (*) were present within the TH‐ir neurons (brown) or their neurites; these TH‐ir neurons were often found surrounded by beaded dopaminergic fibres (**c**,**d**). There were no significant differences in TH‐ir neurons between groups in the agranular and dysgranular subregions (**e**,**f**). Magnification: 630 × , scale bar: 50 μm.

TH‐ir interneurons were predominantly present in the deeper layers (V–VI) of the agranular and dysgranular subregions of the insular cortex and occasionally in layer III and white matter. The granular insular cortex did not contain TH‐ir neurons. These neurons were unipolar, bipolar, tripolar and multipolar. They were also usually surrounded by a mesh of beaded dopaminergic fibres. Assessment of TH and α‐synuclein did not reveal any colocalization. Furthermore, there were no significant differences in the density of TH‐ir neurons between groups for both agranular and dysgranular subregions (*P *=* *0.56 and *P *=* *0.82, respectively) (Figure 6). No significant correlation was found between subregional TH‐ir density and α‐synuclein scores.

**Figure 7:**
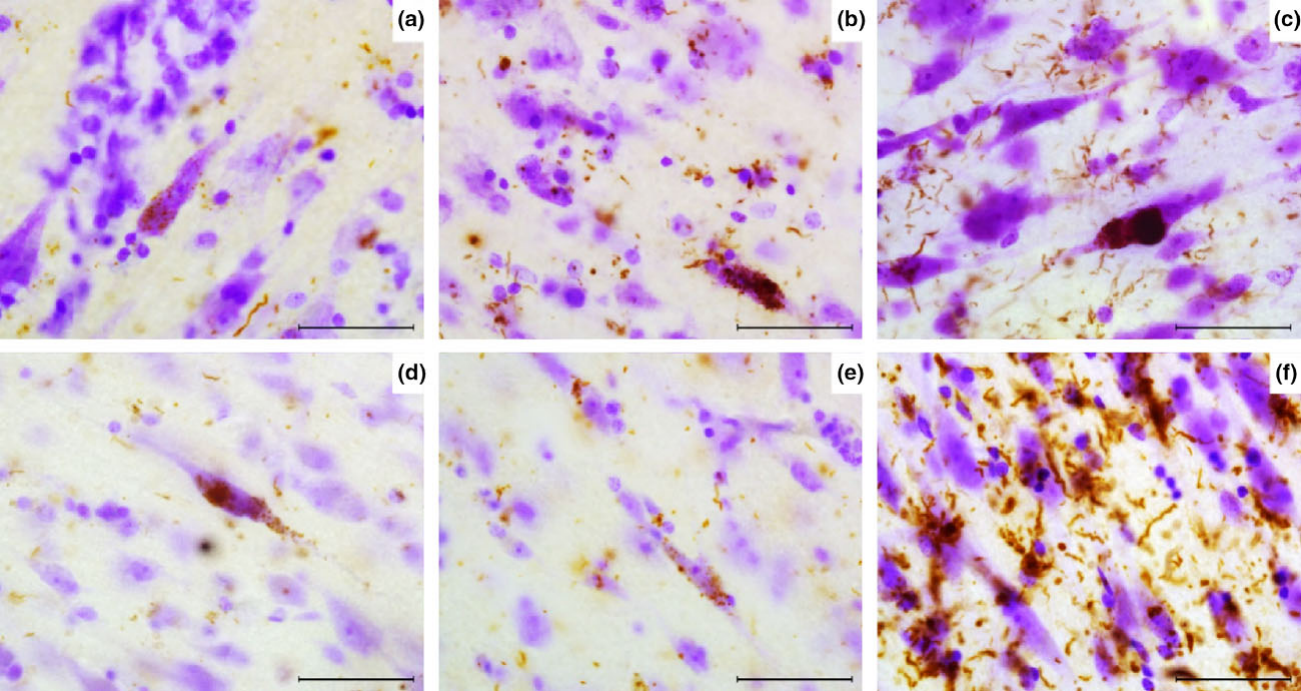
α‐synuclein deposits in VENs. (**a**) PD‐2 shows granular LB inclusions (brown) along a VEN. (**b**) LB in VEN and surrounding astrocytes in PD‐2. (**c**) PDD‐2 shows a VEN containing a large LB and multiple granular inclusions within the cell body, astroglial α‐synuclein inclusions are also seen. (**d**) PDD‐1 shows LB in the soma and dendrite of a VEN. (**e**) α‐synuclein inclusions are shown in a fork cell in PDD‐1. (**f**) DLB‐2 agranular insula shows many deposits surrounding pyramidal neurons and rod shaped VEN. Magnification: 630 × , scale bar 50 μm. PDD, Parkinson's disease dementia; LB, Lewy bodies; DLB, dementia with Lewy bodie; VENs, von Economo neurons.

Only few VENs and fork cells in layer V of fronto‐insular region revealed α‐synuclein inclusions in PD‐2 and PDD 1 and 2. VENs showed granular cytoplasmic α‐synuclein inclusions and LBs. They were also frequently surrounded by astroglial cells showing thorn‐shaped α‐synuclein immunoreactivity (Figure 7).

### Relationship between astrocytes and α‐synuclein pathology

**Figure 8:**
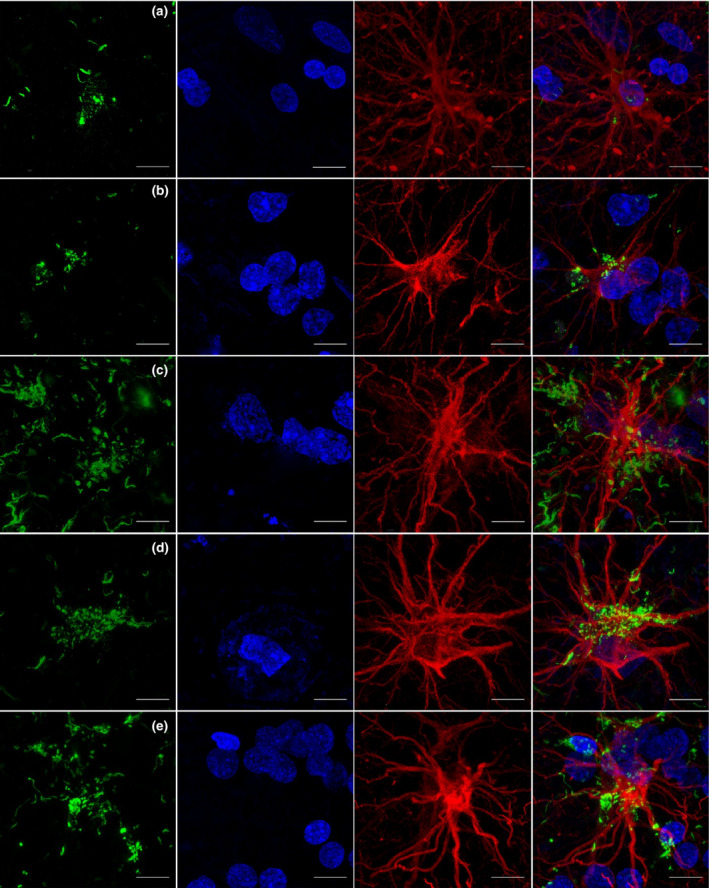
Relationship between α‐synuclein immunoreactivity and astrocytes in insular cortex in PD(D) and DLB. α‐synuclein (green) is present within varicose projection astrocyte (GFAP, red) cell body in deep layer in the anterior insula in PD‐2 (**a**). In PDD‐2, an astrocyte is shown containing α‐synuclein aggregates and surrounded by a cluster of nuclei in the anterior insula (**b**). In DLB‐3, a protoplasmic astrocyte is shown surrounded by multiple α‐synuclein aggregates but no inclusions were present within the astrocyte (**c**). DLB‐1 shows α‐synuclein deposits surrounding the cell body of an interlaminar astrocyte in layer I (**d**) and similar inclusions are shown within a protoplasmic astrocyte, its processes, and the surrounding clustered nuclei in the posterior insula in DLB‐2 (**e**). Magnification 100 × , Scale bar: 10 μm. DLB, dementia with Lewy bodie; PDD, Parkinson's disease dementia; GFAP, glial fibrillary acidic protein.

Double labelling of GFAP and α‐synuclein was performed to examine the relationship between astrocytes and α‐synuclein inclusions. Astrocytes with multiple varicosities were predominantly found in the agranular and to a lesser extent in the dysgranular insula, in both controls and patients, possibly representing varicose projection astrocytes (VPA). A VPA in the infragranular layer in PD‐2 was found containing a cluster of cytoplasmic α‐synuclein forming a mesh‐like structure (Figure 8 **a**). Other protoplasmic and interlaminar astrocytes examined in PDD and DLB showed α‐synuclein deposits around the cell body and processes (Figure 8). Further colocalization analysis in VPA showed a positive correlation between α‐synuclein and GFAP (MOC for channels A and B = 0.94 and 0.27, repectively) (Figure S2). Analysis and reconstruction indicate possible compartmentalization of the α‐synuclein within the astrocytic cell body (Figure S2).

## Discussion

In this case series, we observed a decreasing gradient in the load of α‐synuclein immunoreactivity from the anterior periallocortical agranular subregion to the intermediate pro‐isocortical dysgranular and posterior isocortical granular insula in iLBD, PD and DLB subjects. This was particularly evident in iLBD and PD(D) with the highest load of neuropathological inclusions in the agranular insula. In DLB with high AD pathological stages, there was also extensive α‐synuclein immunoreactivity in the granular region with an abundance of LBs in the infragranular layers V/VI, and relative sparing of layers III/IV. Some VENs, but not TH‐ir neurons, in the anterior insula revealed α‐synuclein inclusions in PD(D). Astrocytes were also vulnerable to α‐synuclein inclusions and showed degenerative changes at all disease stages, yet most prominent in PDD and DLB.

The presence of a gradient for α‐synuclein pathology across the insular cortex, from the anterior agranular to posterior granular subregions, has previously been documented for NFT and β‐amyloid pathology in post mortem insular tissue of AD patients 45. This decreasing pathological gradient appears to be consistent with differences in cytoarchitecture, cell types and myelination in the insular subregions 28. Accordingly, the agranular insula comprises the highest density of acetylcholinesterase and lowest density of myelinated fibres while the opposite exists in the granular subregions 46. The vulnerability of the agranular insula relative to the late and sparse involvement of the granular insula corresponds with an inverse relationship between myelination and neuropathological lesions in both AD and PD 47. The agranular insula also comprises of preferential connections to the olfactory and rhinal cortices which are affected in the early stages of PD and show a similar allocortical cytoarchitecture 18, 29. In line with this, the granular insula connects to the isocortical somatosensory and cingulate cortices and is affected in later stages of the disease 29. Considering the insular phylogenetic and ontogenetic variations 48, the insular subregions reflect the global regional involvement in PD, as described by Braak and colleagues. Moreover, cognitive and neuropsychiatric deficits were prominent in our cohort and particularly with more advanced Braak stages. Considering the anterior insular cortex connectivity, α‐synuclein pathology and cell death in the agranular insula may contribute to autonomic, cognitive and psychiatric symptoms in PD(D) and DLB 49.

TH‐ir neurons ranged from bipolar to multipolar, predominantly resided in the deeper layers of the agranular and dysgranular insular cortex subregions and did not show any α‐synuclein deposits. Generally, catecholaminergic neurons in the brain stem are known to be selectively vulnerable to Lewy pathology in PD 50. Yet, cortical TH‐ir neurons remain mysterious and show substantial differences in distribution pattern across the brain. Although the lowest density is present within the somatosensory cortex, the highest is present in the cingulate cortex 11. This variation is also represented by the insula with a decreasing gradient of TH‐ir neurons from agranular/dysgranular to granular/hypergranular insula, adding to the variation in cellular compositions across the insular subregions. Although a previous study showed reduction in TH‐ir neurons in PD compared to controls in multiple cortical brain regions 10, we did not find a significant reduction, which may be the result of a limited sample size. We also show that VENs are vulnerable to α‐synuclein pathology in advanced PD(D). VENs have been implicated in consciousness, emotion, cognition and social awareness 31, 43, 51. In this study, few VENs showed α‐synuclein inclusions relative to the greater involvement of pyramidal neurons within the agranular insula. Previous studies assessing VENs found hyperphosphorylated tau inclusions as well as significant cell loss in Pick's disease compared to AD 52, 53. Furthermore, VENs are known to be selectively vulnerable to degeneration early in frontotemporal dementia as well as early onset schizophrenia 31. Other studies, however, have shown that VENs show a reduced density as well as NFT in AD particularly in late stages of the disease compared to cognitively normal elderly controls and super‐agers, elderly who performed average for their age group or above average for individuals in their 50s and 60s, repectively 54, 55. VENs are uniqe spindle shaped projection neurons with sparse dendritic branching. They are therefore thought to function in the rapid relay of inputs from the insula and anterior cingulate cortex to other brain regions. This in turn would allow rapid control of behavior during changing social situations 56. Recent biochemical analyses showed that these neurons may also possess a novel type of cortical monoaminergic function due to their expression of VMAT2 which packages monoamines into vesicles 57. Moreover, assessment of the functional connectivity of regions containing VENs showed their involvement in networks involved in salience processing, allowing for rapid relay of information to other brain regions and thus controlling attention 58. The salience network, formed of anterior ventral frontoinsular region and anterior cingulate cortex, is presumed to play a role in detecting salient stimuli and directing attention to such stimuli by coordinating between other brain networks to facilitate a goal‐directed behaviour 59, 60. The salience network has been implicated in PD where patients showed reduced dopaminergic receptors within the network which consequently could play a role in memory and executive dysfunctions 61. Despite previous implications on the possible role of VENs in PD, our study is the first to show their involvement in PD(D) and DLB. Future studies focusing on the loss of VENs in Lewy body diseases may provide some insight into their contribution to autonomic and neuropsychiatric symptoms.

Other cells such as astrocytes have been previously shown to contain α‐synuclein inclusions in advanced PD and parallel to the neuronal involvement in disease 42. However, minimal astrocytic activation and cytoplasmic α‐synuclein inclusions were observed in PD compared to other neurodegenerative diseases 62. In our series, we observed the enwrapment of astrocytic cell bodies and processes with α‐synuclein. Protoplasmic and interlaminar astrocytes showed extensive synucleinopathy most severe in the agranular insula, particularly in PDD with glial tauopathy (ARTAG). However, it remains unknown what role astroglial α‐synuclein plays in disease progression and its relationship with other pathologies. Moreover, whether astroglial tauopathy could play a role in the vulnerability of astroglia to synucleinopathy and degeneration remains unclear 63, 64. Another novel feature of the agranular insula, is the presence of VPA. These recently discovered astrocytes with varicosities along their processes were found only in higher order primates and humans 65. Recent studies proposed that they may provide alternative pathways for long distance communication through cortical layers 65, 66. We report the presence of intracellular α‐synuclein inclusions within these VPA cells. Future studies studying these cells in more detail may provide more insight into their selective vulnerability and functional correlates in PD and DLB. This is the first study to assess the subregional neuropathological characteristics and selective vulnerability of the insular cortex in PD(D) and DLB. The limitations of this study include a limited sample size. Our study also does not include quantitative data on the density of VENs in PD and DLB. Attributable to the presence of VENs primarily within the agranular insula and particularly perpendicular to the pia, it requires a dissection approach different from that used in the present study 53. Future large clinicopathological studies including longitudinal data and insular subregional analysis will aid in our understanding of the impact of insular neurodegeneration on the cognitive and psychiatric deficits in PD and DLB.

In conclusion, the distribution pattern of α‐synuclein pathology revealed a decreasing anterior‐to‐posterior gradient in the insular cortex, representative of the differential cytoarchitectural vulnerability in PD and DLB. Our study also shows that VENs and astroglia are vulnerable to α‐synuclein pathology, particularly in advanced Braak stages in PDD and DLB. These results elucidate variations in the selective vulnerability of neurons and astrocytes as well as the pathological distribution pattern between the allocortical and isocortical subregions of the insular cortex.

## Author contributions

The study design was carried out by WvB and YF. YF carried out the experimental work and wrote the initial manuscript. WvB and AR performed the autopsies and WvB, AR and YF completed the diagnostic scoring. AJ and EO provided technical help and EO performed analysis of TH cell density in the study. AD provided technical advice and revised the manuscript. FJ participated in the design and revised the manuscript. Significant contributions were provided by WvB, AD, AR, AJ, EO and FJ and the manuscript was then edited and finalized by YF and WvB.

## Ethical approval

The procedures of the Netherlands Brain bank have been approved by local ethical committee, VUmc Amsterdam.

## Disclosures

The authors declare having no conflict of interest.

## Supporting information


**Figure S1.** Median α‐synuclein pathology scores within the insular subregions in PD, PDD and dementia with Lewy bodies (DLB) patient groups.
**Figure S2.** Colocalization of astrocytes [glial fibrillary acidic protein (GFAP)‐red] and α‐synuclein (green).
**Table S1.** Semiquantitative assessment of α‐synuclein pathology in inuslar subregions.
