# Supplementary material for: Differential insular cortex subregional vulnerability to α‐synuclein pathology in Parkinson's disease and dementia with Lewy bodies
Source: Neuropathol Appl Neurobiol. 2018 Jun 26;45(3):262–77. doi: 10.1111/nan.12501 (PMC7380008; doi:10.1111/nan.12501)
Supplement: Supplementary file 1 — Figure S1. Median α‐synuclein pathology scores within the insular subregions in PD, PDD and dementia with Lewy bodies (DLB) patient groups. Figure S2. Colocalization of astrocytes [glial fibrillary acidic protein (GFAP)‐red] and α‐synuclein (green). Table S1. Semiquantitative assessment of α‐synuclein pathology in inuslar subregions. [file NAN-45-262-s001.docx]

**Supplementary**

**Insular cortex sub-region-dependent distribution pattern of α-synuclein immunoreactivity in Parkinson’s disease and dementia with Lewy bodies**

Yasmine Y. Fathy^1^, Allert J. Jonker^1^, Ellen Oudejans^1^, Frank Jan J. de Jong^2^, Anne-Marie van Dam^1^, Annemieke J.M. Rozemuller^3^ , Wilma D.J. van de Berg^1^

^1^ Department of Anatomy and Neurosciences, section Clinical Neuroanatomy, Amsterdam Neuroscience, VU University Medical Center, De Boelelaan 1108, 1081 HZ Amsterdam, Netherlands

^2^Department of Neurology, Erasmus Medical Center, Rotterdam, Postbus 2040 3000 CA, Rotterdam, Netherlands
^3^Department of Pathology, Amsterdam Neuroscience, VU University Medical center, De Boelelaan 1117, 1081 HV Amsterdam, Netherlands

**Corresponding author:**

Yasmine Fathy, MD

Dept. of Anatomy & Neurosciences,

Amsterdam Neuroscience

VU University Medical Center

O2 building

De Boelelaan 1108

1081 HZ Amsterdam, Netherlands

e-mail: y.fathy@vumc.nl; Tel.nr: +31615573540

**Semi-Quantitative Scoring of α-synuclein**

α-Synuclein inclusions were assessed semi-quantitatively using an ordinal scoring system. All layers were assessed by FY and EO at 200x magnification per sub-region and the severity of inclusions was scored from 0-3**.** The scoring criterion was based on the recommendations of the Consensus Diagnostic criteria for DLB with consideration of the tissue thickness. Scoring criteria: 0= absent, 1= few dot-like deposits or sparse LNs present + 1-5 LB, 2= Moderate LN present in all layers + 5-10 LB, 3= Severe LNs present in all layers + >10 LB in 20x objective field. α-Synuclein deposits were counted in all layers and ≥ 3 frames. Median values were then taken for each score. Table S1 provides the α-synuclein semi-quantitative scores for all 3 sub-regions and all subjects.

**Ordinal scores of α-synuclein pathology in insular sub-regions**

| **Patient** | **Agranular score** | **Dysgranular score** | **Granular score** |
| --- | --- | --- | --- |
| iLBD-1 | 2 | 1 | 1 |
| PD-1 | 1 | 1 | 1 |
| PD-2 | 3 | 2 | 1 |
| PD_3 | 2 | 2 | 1 |
| PD_4 | 2 | 1 | 1 |
| PD_5 | 1 | 1 | 0 |
| PDD-1 | 2 | 2 | 1 |
| PDD-2 | 3 | 2 | 1 |
| PDD-3 | 2 | 2 | 2 |
| PDD-4 | 2 | 2 | 1 |
| PDD-5 | 1 | 1 | 1 |
| PDD-6 | 3 | 3 | 2 |
| PDD-7 | 3 | 2 | 2 |
| PDD_8 | 3 | 2 | 2 |
| PDD_9 | 2 | 1 | 1 |
| PDD-10 | 2 | 2 | 2 |
| DLB-1 | 3 | 3 | 3 |
| DLB-2 | 3 | 3 | 3 |
| DLB-3 | 2 | 2 | 1 |
| DLB_4 | 1 | 1 | 1 |
| DLB_5 | 1 | 2 | 1 |

**Table S1**. Semi-quantitative assessment of α-synuclein pathology in inuslar sub-regions. An ordinal scoring system was used for scoring of load of α-synuclein in the insula. 0= absent, 1= few dot-like deposits or sparse LN present +/- 1-5 LB, 2= Moderate LN present in all layers +/- 5-10 LB, 3= Severe LN present in all layers +/- >10 LB. LB: Lewy bodies, LN: Lewy neurites

**Semi-quantitative analysis of the α-synuclein pathology revealed a difference in local density between groups**

The insular cortex showed a decreasing gradient of α-synuclein pathology from anterior to posterior, particularly in PD and PDD. In DLB, patients with a rapid disease progression (DLB-1 and DLB-2) showed a less apparent gradient due to greater α-synuclein scores within the posterior granular insula. Kruskal Wallis analysis to test differences in α-synuclein scores between groups was performed and showed a significant difference between groups (χ2 (2, N=63)=7,602, p=0.022).

**Figure S1. Median α-Synuclein pathology scores within the insular sub-regions in PD, PDD and DLB patient groups.** α-Synuclein pathology was scored based on a semi-quantitative ordinal scoring system ranging from 0-3 representing pathological load from mild to severe. A significant difference in scores between groups was found (χ2 (2, N=63)=7,602, p=0.022). Pairwise comparisons showed a significant difference between the PD and PDD groups (p = 0.036).

**Colocalization between α-synuclein pathology and astrocytes**

Colocalization between was assessed using Imaris 8.3 (Bitplane, South Windsor, CT, USA). A region of interest (ROI) was manually selected for each image and colocalization between GFAP and α-synuclein (BD Biosciences) was analyzed. The Pearson’s correlation coefficient (PCC) and Mander’s overlap coefficient (MOC) were used as a measure of colocalization between the two channels. PCC of colocalized regions showed poor correlation in all cases (**Figure S2.**). In DLB-1, an interlaminar astrocyte in the superficial layers showed a large MOC correlation between α-synuclein and GFAP (0.9781 & 0.6172, respectively) with % of colocalized volume= 7.17 (**a-c**). PD-2 showed an astrocyte with varicosities along its processes(*), possibly a varicose projection astrocyte, analysis of which showed an intracellular meshwork of α-synuclein. Colocalization analysis yielded an inverse PCC in colocalized volume (-0.09) and a large correlation was found between both structures using MOC (0.93 & 0.27) with % of colocalized ROI=1.92 (**d-f**).

**
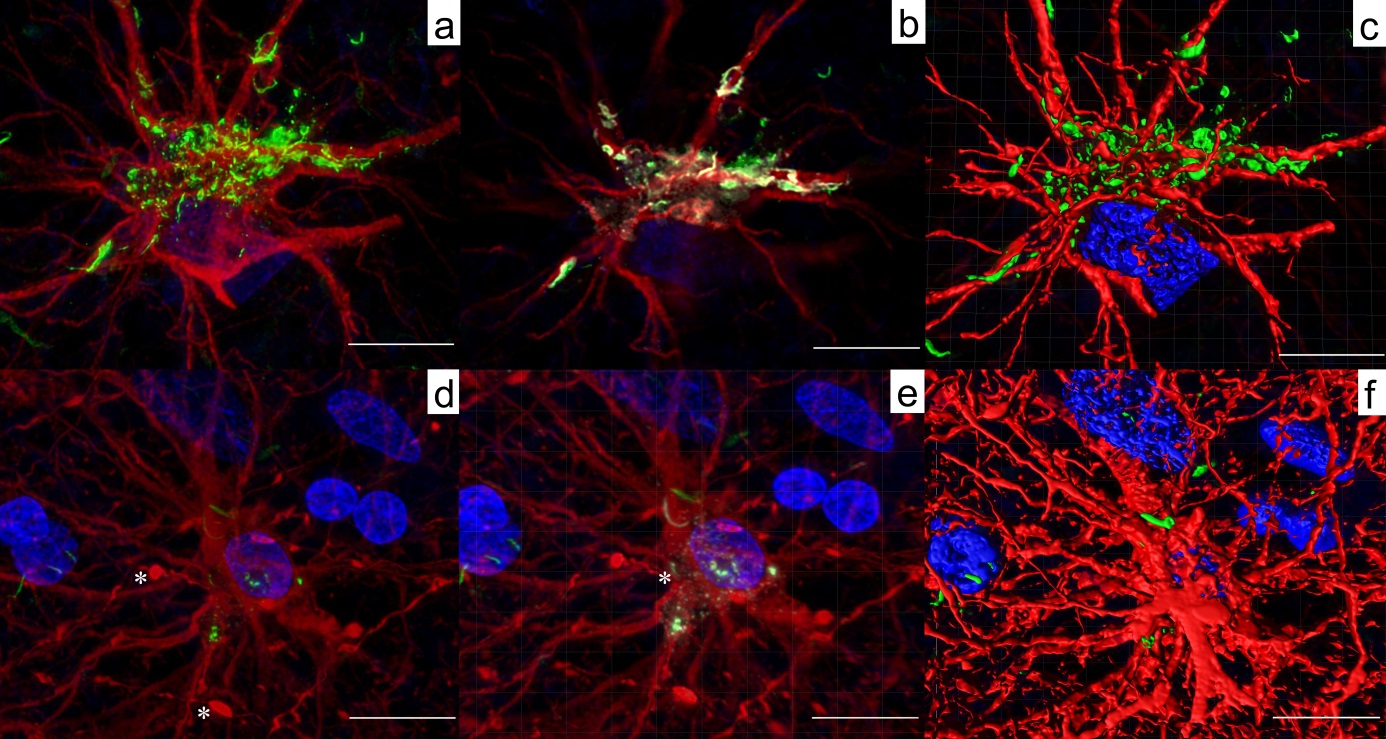
**

**Figure S2.** **Colocalization of Astrocytes (GFAP-red) and α-synuclein (green).** Top Figure: (**a**) Confocal image shows α-synuclein deposits surrounding a GFAP + astrocyte in DLB-2. (**b**) Image of the colocalized ROI illustrates wrapping of the astrocyte by a cluster of α-synuclein. (**c**) Reconstruction of the astrocyte illustrates the relationship between both structures. (**d**) In PD-2 a varicose projection astrocyte is seen (*). (**e**) The colocalized ROI shows an α-synuclein meshwork within the astrocytic cell body (*). (**f**) Reconstruction shows the high degree of branching of the astrocyte while the α-synuclein mesh is hidden within the cell body. MOC: Mander’s overlap coefficient, PCC: Pearson’s Correlation Coefficient. Scale bar: 10µm
